# The diagnostic value of neutrophil to lymphocyte ratio, albumin to fibrinogen ratio, and lymphocyte to monocyte ratio in Parkinson’s disease: a retrospective study

**DOI:** 10.3389/fneur.2024.1450221

**Published:** 2024-09-02

**Authors:** Yi-Ming Li, Xiao-Hu Xu, Li-Na Ren, Xiao-Fan Xu, Yi-Long Dai, Rui-Rui Yang, Cheng-Qiang Jin

**Affiliations:** ^1^Department of Clinical Medicine, Jining Medical University, Jining, Shandong, China; ^2^Medical Laboratory, Affiliated Hospital of Jining Medical University, Jining, Shandong, China; ^3^Neurology Department, Shandong Provincial Hospital Affiliated to Shandong First Medical University, Jinan, China

**Keywords:** Parkinson’s disease, albumin-to-fibrinogen ratio (AFR), disease duration, inflammatory index, disease diagnosis

## Abstract

**Background:**

Parkinson’s disease (PD) is a prevalent disorder of the central nervous system, marked by the degeneration of dopamine (DA) neurons in the ventral midbrain. In the pathogenesis of PD, inflammation hypothesis has been concerned. This study aims to investigate clinical indicators of peripheral inflammation in PD patients and to explore the diagnostic value of neutrophil-to-lymphocyte ratio (NLR), albumin-to-fibrinogen ratio (AFR), and lymphocyte-to-monocyte ratio (LMR) in assessing PD risk.

**Methods:**

This study included 186 patients with PD and 201 matched healthy controls (HC) with baseline data. Firstly, the differences of hematological indicators between PD group and healthy participants were compared and analyzed. Univariate and multivariate regression analyses were then conducted. Smooth curve fitting was applied to further validate the relationships between NLR, LMR, AFR, and PD. Subsequently, subgroup analysis was conducted in PD group according to different duration of disease and Hoehn and Yahr (H&Y) stage, comparing differences in clinical indicators. Finally, the receiver operating characteristic (ROC) curve was employed to assess the diagnostic value of NLR, LMR, and AFR in PD.

**Results:**

Compared to the HC group, the PD group showed significantly higher levels of hypertension, diabetes, neutrophil count, monocyte count, CRP, homocysteine, fibrinogen, and NLR. Conversely, levels of LMR, AFR, lymphocyte count, HDL, LDL, TG, TC, uric acid, and albumin were significantly lower. The multivariate regression model indicated that NLR (OR = 1.79, 95% CI: 1.39–2.31, *p* < 0.001), LMR (OR = 0.75, 95% CI: 0.66–0.85, *p* < 0.001), and AFR (OR = 0.79, 95% CI: 0.73–0.85, *p* < 0.001) were significant factors associated with PD. Smooth curve fitting revealed that NLR was positively linked to PD risk, whereas AFR and LMR were inversely associated with it. In ROC curve analysis, the AUC of AFR was 0.7290, the sensitivity was 63.98%, and the specificity was 76.00%. The AUC of NLR was 0.6200, the sensitivity was 50.54%, and the specificity was 71.50%. The AUC of LMR was 0.6253, the sensitivity was 48.39%, and the specificity was 73.00%. The AUC of the combination was 0.7498, the sensitivity was 74.19%, and the specificity was 64.00%.

**Conclusion:**

Our findings indicate that NLR, LMR, and AFR are significantly associated with Parkinson’s disease and may serve as diagnostic markers.

## 1 Background

Parkinson’s disease (PD) is the second most common neurodegenerative disorder, characterized by the degeneration of dopaminergic neurons, which results in decreased dopamine levels in the striatum (1). The clinical manifestations of Parkinson’s disease include bradykinesia, resting tremor, rigidity, and changes in posture and gait (2). Based on the predominance of specific motor symptoms at diagnosis, the main subtypes of Parkinson’s disease (PD) are: tremor dominant (TD) and postural instability and gait disorder (PIGD) (3). These symptoms are often accompanied by gait disorders, trunk stiffness, poor balance and coordination, and even vocal cord paralysis (4). In addition, patients may experience a variety of non-motor symptoms (NMS), such as decreased sense of smell, constipation, orthostatic hypotension, memory loss, depression, pain, and sleep disturbances (5). Ultimately, at the level of individual behavior, these changes affect an individual’s quality of life.

Both genetic and environmental factors contribute to the etiology of Parkinson’s disease; however, its precise cause remains unknown. Neuroinflammation, a critical factor in neurological disease progression, is a defensive response of the central nervous system to harmful stimuli, triggering protective reactions (6). Upon activation of the inflammatory response, various immune cells secrete an array of cytokines and chemokines into the plasma (7). Substantial evidence currently suggests that inflammation may be an early event and play a crucial role in the development of PD. Although chronic neuroinflammation may not be a initiating factor in Parkinson’s disease, it appears to be a cofactor in disease progression (8, 9).

Multiple preclinical and epidemiological studies have indicated that elevated levels of inflammatory mediators in early PD may contribute to the progressive loss of DA neurons in the substantia nigra (10). Cytokines resulting from oxidative stress and inflammation are associated with the progressive degeneration of nerve conduction pathways. The above evidence indicate that the influence of inflammation in PD cannot be ignored, and that focusing on the inflammatory process in PD patients is important for the development and management of the disease. Therefore, clinically relevant inflammatory indicators in PD warrant attention. Common inflammatory markers in peripheral blood include white blood cells, lymphocytes, neutrophils, and C-reactive protein (CRP). In addition, the neutrophil-to-lymphocyte ratio (NLR), albumin-fibrinogen ratio (AFR) and lymphocyte-to-monocyte ratio (LMR) are emerging as novel inflammatory assessment markers. These markers can not only reflect systemic inflammation but have also been shown to have predictive value in ovarian cancer (11), colorectal cancer (12, 13), chronic lymphocytic leukemia (14) and other diseases. They have been extensively evaluated for their prognostic value in various malignant tumors. Regarding inflammatory parameters, a retrospective study indicated that the systemic inflammatory response index (SII) is an important factor influencing the exercise performance of PD patients. SII may serve as a useful tool for predicting the severity and prognosis of movement disorders in PD patients (15). However, few studies have reported the association of AFR, NLR and LMR with disease severity and their diagnostic value of disease risk in PD patients. Therefore, this study mainly compared the differences in common inflammatory related indicators (white blood cells, lymphocytes, CRP, etc.) and novel inflammatory indicators (NLR, LMR, AFR, etc.) in peripheral blood between healthy individuals and PD patients. Additionally, we explored their diagnostic value for PD.

## 2 Methods

### 2.1 Study population

The patients with PD admitted to the Department of Neurology of the Affiliated Hospital of Jining Medical College between January 2021 and January 2024 were enrolled in this study. This retrospective study analyzed a total of 186 PD patients, comprising 92 females and 94 males. Patients were enrolled in the study conformed to the diagnostic criteria established by the International Parkinson’s Disease Association (16). The exclusion criteria are as follows: (1) atypical PD or other central nervous system diseases, cancer; (2) severe liver or kidney impairment; (3) history of immune suppression or anti-inflammatory treatment; (4) acute infection; (5) autoimmune disease; and (6) patients with significant clinical emotional disorder who are unable to cooperate with clinical evaluation. The healthy control group (HC) consisted of 201 individuals from the health examination center of our hospital during the same period, matched for age and gender. The study was approved by the Ethics Committee at the Affiliated Hospital of Jining Medical College.

### 2.2 Clinical participants information collection

We have collected basic information from all subjects, including age, gender, BMI, smoking history, alcohol history, past medical history, etc. Laboratory examination data primarily includes routine blood tests and blood biochemistry results. Based on Hoehn–Yahr staging, patients were classified into early stage (1–2.5) and medium-advanced stage (≥3). In addition, we calculated the AFR, NLR, and LMR for each patient. AFR = albumin divided by fibrinogen; NLR = absolute neutrophil count divided by absolute lymphocyte count; LMR = absolute lymphocyte count divided by absolute monocyte count.

### 2.3 Statistical analyses

If the continuous variable follows a normal distribution, it is represented by the mean ± SD deviation. Otherwise, the median (quartile) is utilized. Normally distributed data were analyzed using the *t*-test, while non-normally distributed data were assessed using the Mann–Whitney U test and Kruskal–Wallis test to determine statistical significance between groups. Univariate and multivariate regression analyses were conducted to explore the relationship between statistically significant variables and PD. Further, smooth curve fitting was employed to describe the relationship between variables and PD. In order to observe the diagnostic performance of clinical indicators of PD, receiver operating characteristic curve (ROC) was generated. For all statistical analyses, data were analyzed with the use of the statistical packages R (The R Foundation; http://www.r-project.org; version 4.2.0) and EmpowerStats (www.empowerstats.net, X&Y solutions, Inc. Boston, Massachusetts). All *p* values were two-sided, and those less than 0.05 were considered statistically significant.

## 3 Results

### 3.1 Comparison of baseline demographic characteristics and laboratory indexes between PD group and HC group

Table 1 compares baseline data between the healthy control (HC) group and the Parkinson’s disease (PD) group. The age of the HC group was 68 years (66–75) and that of the PD group was 72 years (66–77). The PD group comprised 92 males (49.46%) and 94 females (50.54%). The average BMI of HC group and PD group was 23.45 and 23.63, respectively. There was no significant differences in age, sex and BMI between the two groups (*p* > 0.05). Therefore, the data of the two groups were comparable. The HC group had a lower prevalence of smoking and alcohol consumption compared to the PD group. The PD group had a significantly higher number of patients with hypertension and diabetes compared to the HC group (*p* < 0.05). In laboratory tests, the PD group had higher neutrophil count, monocyte count, CRP, homocysteine, fibrinogen, and NLR levels than the HC group, with statistically significant differences (*p* < 0.05). The HC group had higher platelet count, lymphocyte count, HDL, LDL, TG, TC, uric acid, serum albumin, LMR and AFR levels, and the differences were statistically significant (*p* < 0.05). Additionally, the PD group had slightly higher WBC levels, but this difference was not statistically significant.

**Table 1:** Characteristics of PD patients and healthy controls.

Clinical parameters	HC (*n* = 200)	PD patients (*n* = 186)	*p*-value
AGE	68.00 (66.00–75.00)	72.00 (66.00–77.00)	0.991
Gender (*n*, %)			0.06
Male	118 (59.00%)	92 (49.46%)	
Female	82 (41.00%)	94 (50.54%)	
BMI	23.45 ± 2.84	23.63 ± 3.27	0.565
Hypertension (*n*, %)	12 (6.00%)	76 (40.86%)	<0.001
Diabetes status (*n*, %)	192 (96.00%)	149 (80.11%)	<0.001
Smoking status (*n*, %)			0.006
Yes	13 (6.50%)	28 (15.05%)	
No	187 (93.50%)	158 (84.95%)	
Alcohol consumption (*n*, %)			<0.001
Yes	7 (3.50%)	27 (14.52%)	
No	193 (96.50%)	159 (85.48%)	
WBCs (×10^9^/L)	5.69 ± 1.37	5.99 ± 1.88	0.071
Platelets (×10^12^/L)	243.00 ± 65.43	218.42 ± 69.03	<0.001
Lymphocytes (×10^9^/L)	1.80 ± 0.50	1.64 ± 0.62	0.005
Neutrophils (×10^9^/L)	3.33 ± 1.16	3.74 ± 1.64	0.005
HDL-C (mmol/L)	1.39 ± 0.27	1.30 ± 0.31	0.003
LDL-C (mmol/L)	3.02 ± 0.82	2.45 ± 0.85	<0.001
Monocytes (×10^9^/L)	0.35 ± 0.11	0.40 ± 0.17	<0.001
TG (mmol/L)	1.54 ± 0.81	1.14 ± 0.51	<0.001
TC (mmol/L)	4.89 ± 1.01	4.02 ± 1.01	<0.001
Serum uric acid (μmol/L)	305.31 ± 74.70	245.31 ± 84.14	<0.001
CRP (mg/L)	2.36 ± 2.82	8.76 ± 19.36	<0.001
Homocysteine (mmol/L)	8.48 ± 5.77	13.31 ± 7.49	<0.001
Fibrinogen (g/L)	3.01 ± 0.68	3.27 ± 1.50	0.028
Serum albumin (g/L)	46.97 ± 2.49	40.50 ± 4.54	<0.001
NLR (×109/mmol)	1.97 ± 0.85	2.70 ± 2.23	<0.001
LMR (×109/mmol)	5.55 ± 2.15	4.62 ± 2.13	<0.001
AFR	16.30 ± 3.35	13.46 ± 3.60	<0.001

BMI, body mass index; WBCs, white blood cells; HDL-C, high-density lipoprotein cholesterol; LDL-C, low density lipoprotein cholesterol; TG, triglyceride; TC, total cholesterol; CRP, C-reactive protein; NLR, neutrophil-lymphocyte ratio; AFR, albumin-fibrinogen ratio; LMR, lymphocyte-monocyte ratio.

### 3.2 Univariate and multivariate regression analyses

In the univariate analysis presented in Table 2, we have identified several influencing factors of Parkinson’s disease (PD), including smoking, alcohol consumption, hypertension, diabetes, platelet count, lymphocyte count, neutrophil count, monocyte count, HDL, LDL, TG, TC, uric acid, CRP, homocysteine, fibrinogen, serum albumin, NLR, LMR, and AFR. After adjusting for confounding variables such as body mass index, uric acid levels, total cholesterol levels, and triglyceride levels, the multivariate regression model revealed that NLR (OR = 1.79, 95% CI: 1.39–2.31, *p* < 0.001), LMR (OR = 0.75, 95% CI: 0.66–0.85, *p* < 0.001), and AFR (OR = 0.79, 95% CI: 0.73–0.85, *p* < 0.001) were significant factors associated with PD (Table 3).

**Table 2:** The influencing factors analysis of PD.

Variables	OR (95% CI)	*p*-value
Smoking	2.55 (1.28, 5.09)	<0.001
Alcohol consumption	4.68 (1.99, 11.04)	<0.001
BMI	1.02 (0.95, 1.09)	0.564
Hypertension	10.82 (5.64, 20.79)	<0.001
Diabetes	5.96 (2.70, 13.18)	<0.001
Platelets	0.99 (0.99, 1.00)	<0.001
Lymphocytes	0.59 (0.41, 0.86)	<0.001
Neutrophils	1.24 (1.06, 1.44)	0.007
HDL-C	0.34 (0.17, 0.70)	0.003
LDL-C	0.44 (0.33, 0.57)	<0.001
Monocytes	14.28 (2.92, 69.92)	<0.001
TG	0.37 (0.25, 0.53)	<0.001
TC	0.42 (0.33, 0.54)	<0.001
Serum uric acid	0.99 (0.99, 0.99)	<0.001
CRP	1.09 (1.04, 1.14)	<0.001
Homocysteine	1.18 (1.12, 1.24)	<0.001
Fibrinogen	1.37 (1.04, 1.82)	0.027
Serum albumin	0.55 (0.49, 0.62)	<0.001
NLR	1.56 (1.27, 1.93)	<0.001
LMR	0.81 (0.74, 0.90)	<0.001
AFR	0.78 (0.73, 0.84)	<0.001

BMI, body mass index; WBCs, white blood cells; HDL-C, high-density lipoprotein cholesterol; LDL-C, low density lipoprotein cholesterol; TG, triglyceride; TC, total cholesterol; CRP, C-reactive protein; NLR, neutrophil-lymphocyte ratio; AFR, albumin-fibrinogen ratio; LMR, lymphocyte-monocyte ratio.

**Table 3:** Multivariate regression analyses for PD.

Variable	OR	95%CI	*p*-value
NLR	1.79	(1.39, 2.31)	<0.0001
LMR	0.75	(0.66, 0.85)	<0.0001
AFR	0.79	(0.73, 0.85)	<0.0001

NLR, neutrophil-lymphocyte ratio; LMR, lymphocyte-monocyte ratio; AFR, albumin-fibrinogen ratio.

### 3.3 Smooth curve fitting

**Figure 1:**
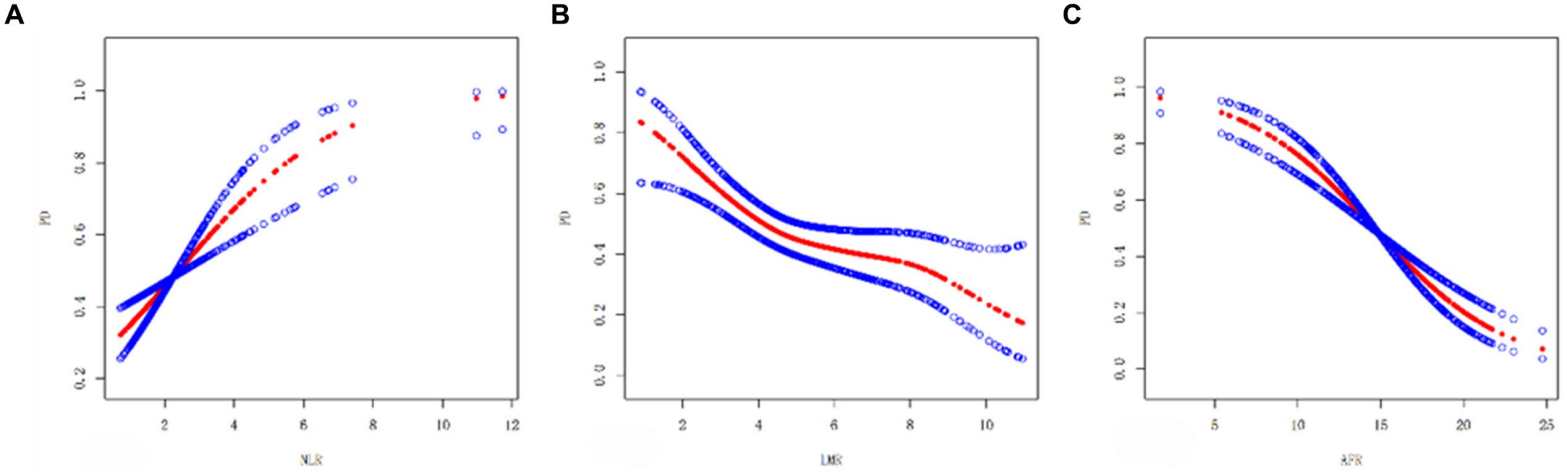
Smooth curve plots **(A–C)**: **(A)** Smooth curve between NLR and PD. **(B)** Smooth curve of LMR and PD. **(C)** Smooth curve of AFR and PD. The red dashed line shows the spline plot of PD, and the blue dashed line shows the 95% CI of the spline plot.

A smooth curve fitting chart is shown in Figure 1. As shown in Figure 1A, NLR positively associated with PD risk, LMR, AFR negatively associated with PD risk (Figures 1B,C).

### 3.4 Analysis stratified by course of PD disease

The impact of disease duration on PD cannot be overlooked. The PD population was further categorized based on disease duration into ≤2 years, 3–6 years, and >6 years. We compared the blood indicators of PD patients among different durations. Table 4 presents a comparison of data for PD patients categorized by duration. The results indicated that as PD duration increased, white blood cell count and serum uric acid (UA) levels gradually decreased in PD patients, although the differences were non-significant (*p* > 0.05). Lymphocyte count decreased significantly (*p* < 0.05) in PD patients with a duration of >6 years, demonstrating significant differences between groups. Meanwhile, NLR and MONO levels were significantly higher in the intermediate duration group (3–6 years) compared to the short duration group (≤2 years). In the intermediate duration (3–6 years), the LMR level was lower than that in the low-course group, while the MONO level was significantly higher than that in the high-course group (*p* < 0.05).

**Table 4:** PD patients with different course of disease.

	≤2 years (*n* = 86)	3–6 years (*n* = 53)	>6 years (*n* = 47)	*p*-value
WBCs (×10^9^/L)	5.91 (4.75–6.88)	5.52 (4.84–6.85)	5.40 (4.62–7.29)	0.802
Platelets (×10^12^/L)	213.43 ± 63.62	236.00 ± 88.95	207.74 ± 47.15	0.081
Lymphocytes (×10^9^/L)	1.71 (1.33–2.06)^ab^	1.48 (1.23–1.91)^c^	1.36 (0.95–1.90)	0.014*
Neutrophils (×10^9^/L)	3.42 (2.59–4.09)	3.38 (2.96–4.09)	3.51 (2.63–4.92)	0.470
HDL-C (mmol/L)	1.29 ± 0.30	1.27 ± 0.31	1.35 ± 0.31	0.393
LDL-C (mmol/L)	2.48 ± 0.83	2.50 ± 0.96	2.32 ± 0.75	0.497
Monocytes (×10^9^/L)	0.38 (0.28–0.46)^ab^	0.40 (0.33–0.47)^c^	0.34 (0.27–0.40)	0.041*
TG (mmol/L)	1.04 (0.78–1.39)	1.07 (0.80–1.36)	0.99 (0.80–1.21)	0.341
TC (mmol/L)	4.07 ± 1.02	3.96 ± 1.11	3.99 ± 0.91	0.818
Serum uric acid (μ mol/L)	248 (186–297.00)	247 (201–286)	226 (176–273)	0.755
CRP (mg/L)	1.34 (0.52–5.00)	2.10 (0.63–6.87)	2.31 (0.58–8.84)	0.654
Homocysteine (mmol/L)	11.20 (9.65–13.52)	11.60 (9.90–16.80)	12.10 (10.80–13.95)	0.579
Fibrinogen (g/L)	3.00 (2.70–3.40)	3.20 (2.80–3.40)	3.10 (2.60–3.70)	0.283
Serum albumin (g/L)	41.15 (38.12–43.58)	40.70 (37.90–43.00)	41.30 (37.90–43.70)	0.961
NLR	1.94 (1.46–2.56)^b^	2.29 (1.71–3.14)	2.42 (1.47–3.44)	0.055
LMR	4.77 (3.18–6.44)^ab^	4.05 (2.77–4.77)^c^	4.56 (3.21–5.71)	0.027*
AFR	13.83 (11.13–15.60)	13.05 (11.62–14.89)	13.39 (10.71–15.45)	0.872

^a^≤2 years vs. 3–6 years, *p* < 0.05; ^b^≤2 years vs. >6 years, *p* < 0.05; ^c^3–6 years vs. >6 years, *p* < 0.05. ^*^≤2 years vs. 3–6 years vs. >6 years, *p* < 0.05.

### 3.5 Analysis stratified by PD stage

According to Hoehn–Yahr scale grading, patients were divided into early disease (H&Y grade 1–2.5, *n* = 131) and moderate-late disease (H&Y grade ≥ 3, *n* = 55) groups. There were no statistically significant differences between the two groups in leukocytes, HDL, LDL, monocytes, TG, TC, CRP, uric acid, albumin, neutrophils, homocysteine, NLR, LMR, and AFR levels (Table 5). Compared to early PD patients, those in the advanced stage group had significantly lower platelet levels, lymphocyte counts, and monocyte levels (*p* < 0.05).

**Table 5:** Comparison of laboratory examination in PD patients with early-stage and those with advanced-stage.

	Early stage (*N* = 131)	Advanced stage (*N* = 55)	*p*-value
WBCs (×10^9^/L)	5.91 (4.71–7.07)	5.40 (4.76–6.78)	0.291
Platelets (×10^12^/L)	225.60 ± 75.69	201.35 ± 45.95	0.028
Lymphocytes (×10^9^/L)	1.67 (1.30–2.06)	1.38 (0.95–1.87)	0.032
Neutrophils (×10^9^/L)	3.40 (2.62–4.27)	3.42 (2.72–4.28)	0.074
HDL-C (mmol/L)	1.31 ± 0.30	1.29 ± 0.32	0.746
LDL-C (mmol/L)	2.50 ± 0.89	2.32 ± 0.73	0.191
Monocytes (×10^9^/L)	0.39 (0.29–0.46)	0.35 (0.28–0.40)	0.017
TG (mmol/L)	1.06 (0.78–1.35)	0.97 (0.78–1.23)	0.368
TC (mmol/L)	4.06 ± 1.06	3.92 ± 0.90	0.391
Serum uric acid (μ mol/L)	247 (188–290)	224.00 (185–282)	0.452
CRP (mg/L)	1.74 (0.58–5.00)	1.62 (0.48–8.50)	0.865
Homocysteine (mmol/L)	11.20 (9.60–14.55)	12.00 (10.80–13.75)	0.801
Fibrinogen (g/L)	3.10 (2.70–3.45)	3.10 (2.60–3.40)	0.353
Serum albumin (g/L)	41.30 (38.25–43.35)	40.30 (37.25–43.40)	0.309
NLR	2.10 (1.49–2.79)	2.38 (1.56–3.24)	0.136
LMR	4.28 (3.09–5.75)	4.60 (3.21–5.55)	0.638
AFR	13.50 (10.94–15.43)	13.56 (11.15–16.27)	0.361

### 3.6 Analysis of ROC curve

**Figure 2:**
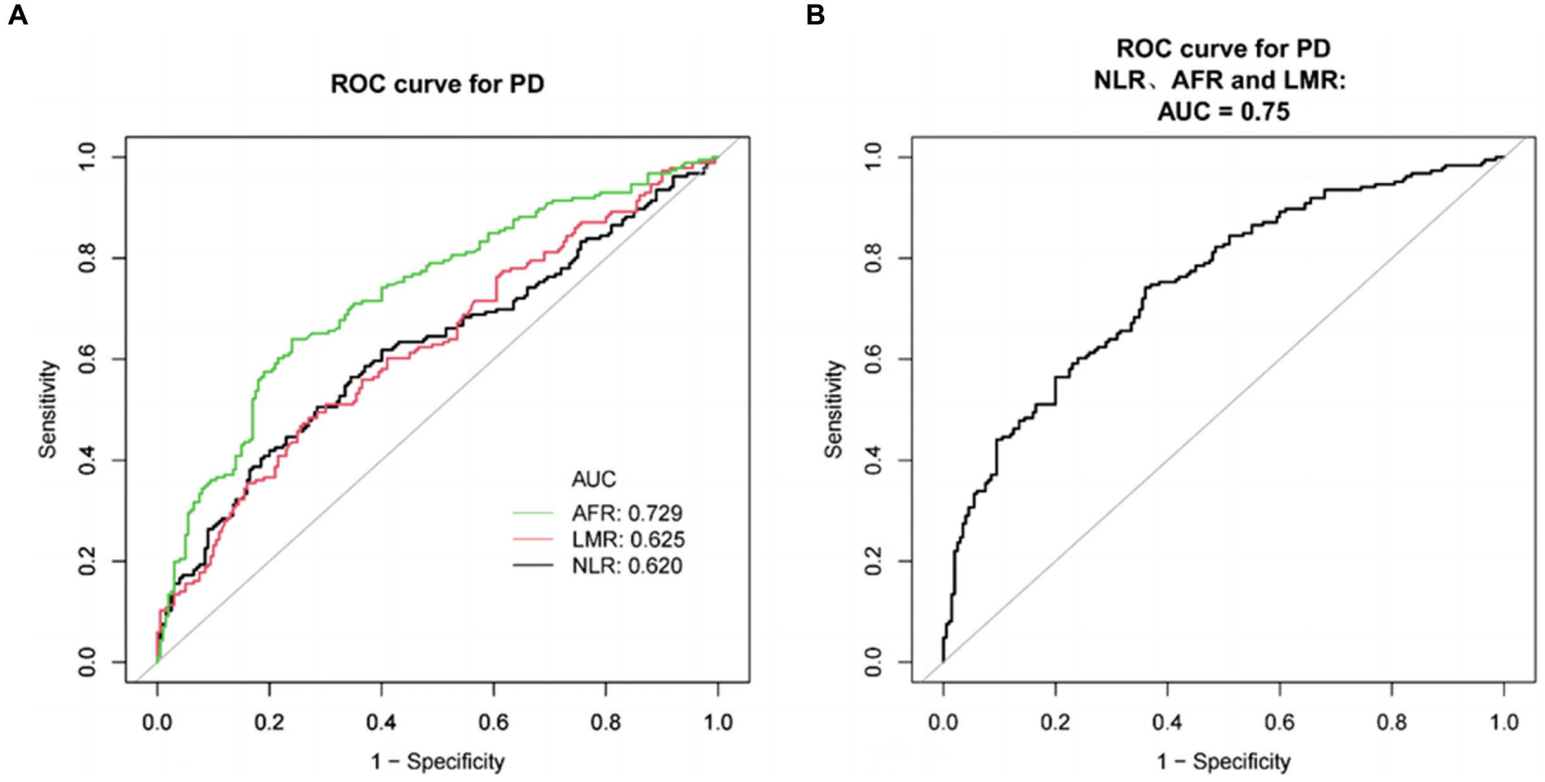
ROC curves for evaluating the efficacy of NLR, LMR, and AFR in PD patients and healthy subjects. **(A)** ROC curve analysis showed that NLR, LMR and AFR had significant PD discrimination ability, and AUC values were 0.6200, 0.6253, and 0.7290, respectively. ROC curve of NLR, LMR and AFR combined detection. **(B)** ROC curve of NLR, LMR, and AFR combined detection. The AUC values was 0.7498.

ROC curve analysis was used to evaluate the predictive values of NLR, LMR, and AFR for PD (Figure 2A). The AUC of AFR was 0.7290, the optimal cutoff value was 14.3831 × 10^9^/mmol, sensitivity of 63.98% and specificity of 76.00%. The AUC of NLR was 0.6200, with a best cutoff value of 2.1822 × 10^9^/mmol, sensitivity of 50.54%, and specificity of 71.50%. The AUC of LMR was 0.6253, the best cut-off value was 4.2989 × 10^9^/mmol, the sensitivity was 48.39%, and specificity was 73.00%. Combining NLR, LMR, and AFR for PD detection resulted in an AUC of 0.7498, with a sensitivity of 74.19% and specificity of 64.00% (Figure 2B).

## 4 Discussion

As for the relationship between inflammation and PD, it was first observed by McGeer in 1988 that activated microglia and infiltrating lymphocytes were present in the substantia nigra of PD patients. This observation led to the hypothesis that inflammation might influence PD (17). In the central nervous system (CNS), the immune defense system is composed of microglial cells, oligodendroglia cells, and astrocytes (18). In cases of infection or injury, cytokines and chemokines can enhance BBB permeability, permitting the entry of peripheral immune cells. Following resolution of acute inflammation, infiltration of the CNS by immune cells can exacerbate chronic inflammation. In PD patients, neuroinflammation relies on the activation of the immune response, which involves a cascade of reactions that produce small molecule proteins and chemokines (19). When the integrity of the BBB may be compromised in PD, it may allow protein aggregates such as α-synuclein to enter the brain and mediate inflammation. As α-synuclein (α-syn) aggregates accumulate in the brain and causing dysfunction of neurons, leading to changes in neural synapses, it becomes one of the mechanisms of PD pathogenesis (20). The inflammation hypothesis is crucial in the pathogenesis of PD. Therefore, assessing inflammation in PD patients is essential for clinical management and prognosis evaluation.

Current clinical research emphasizes assessing systemic inflammation through various biochemical and hematological markers from routine blood tests or derived ratios of these measurements (21). First of all, NLR is the ratio of the relative number of neutrophils to lymphocytes in peripheral blood. Neutrophils are crucial in the first line of defense against external pathogens by initiating and amplifying the inflammatory response. As an easily calculated laboratory biomarker, NLR is useful for assessing systemic inflammation. Previous studies have demonstrated that NLR has different evaluation efficacy for various diseases such as coronary artery disease, hypertension, chronic kidney disease, diabetes, heart failure, cerebrovascular disease, and peripheral artery disease (22). LMR is the lymphocyte-monocyte ratio. Recent studies have revealed the pro-inflammatory effect of α-syn on monocytes in PD patients. Additionally, peripheral mononuclear cells can appear subpopulation shift and be damaged by specific stimuli (23). LMR has been extensively discussed and found to have predictive value in the risk progression of various diseases such as breast cancer, colorectal cancer, lung cancer (24, 25). A large prospective cohort study found associations between NLR, LMR, and PLR with 17 different cancer sites, highlighting their role in systemic inflammation and cancer risk (21). Several clinical studies have confirmed the association of peripheral immune markers with Parkinson’s disease. Specifically, participants with higher counts of immune cells, such as lymphocytes, have a lower likelihood of developing PD (26, 27). A large-scale study from the UK Biobank also found that NLR is a risk factor for PD, while LMR acts as a protective factor, supporting our findings (28). A study compared NLR levels among different subgroups of idiopathic Parkinson’s disease (IPD). These IPD subtypes include akinetic-rigid (AR-IPD) and tremor-dominant (TD-IPD). The conclusion was that there was no difference in NLR levels between IPD, normal control group and IPD subtype (29). These findings imply potential variations in inflammatory markers across different subtypes of PD. In addition, among various neurodegenerative diseases, a study suggested that patients with progressive supranuclear palsy (PSP) exhibited significantly higher NLR values compared to patients with PD and healthy controls (30). Another study by Natalia Madetko et al. on alpha-synuclein diseases (MSA-P and PD) found significantly elevated NLR and PLR values compared to healthy controls. In PD patients, both NLR and PLR values were notably higher than those in the control group. For MSA-P patients, only NLR showed a significant increase (31). Investigating non-specific inflammatory parameters across different neurological diseases is particularly crucial, as these parameters may serve as markers for distinguishing between diseases.

This study not only examined the relationship between NLR, LMR, and the risk of PD but also explored the effect of AFR on PD. The albumin-to-fibrinogen ratio (AFR) is a sensitive parameter reflecting systemic inflammation (12). Serum albumin levels reflect nutritional status and inflammation, with lower levels impairing immune response. Regarding AFR, there is growing evidence that it is an influential regulator of the progression of several malignant tumours and of the systemic inflammatory response (32). Lu and colleagues assessed the prognosis of PD patients undergoing deep brain stimulation (DBS) using the fibrinogen-to-albumin ratio (FAR) and found that high FAR levels were independently associated with postoperative delirium after DBS surgery (33). However, no other study has explicitly proposed a relationship between AFR and PD. This study is the first to propose the relevance and diagnostic value of AFR in PD. Our findings indicate that AFR is a significant factor in PD, with higher AFR levels associated with a decreased likelihood of PD. The interpretation of this result is that fibrinogen may have a direct effect on CNS cells (e.g., neurons, oligodendrocytes, etc.) while participating in the processes of coagulation, inflammation and tissue repair. Fibrinogen is not detected in healthy central nervous system, it can be observed in various neurodegenerative diseases due to the breakdown of the BBB, resulting in its massive deposition (34). Consequently, high fibrinogen deposition in PD patients correlates with lower nutritional status compared to healthy individuals, leading to decreased albumin levels and altered AFR levels. Furthermore, the impact of fibrinogen on PD is controversial, with a Mendelian randomization study suggesting no evidence supporting the association between fibrinogen and γ-fibrinogen levels and PD (35). In contrast, a longitudinal study of Japanese-American men from the Honolulu Heart Study found that high fibrinogen levels were associated with an increased risk of PD in men over 75 years old (36). Due to the limitations of observational studies, further research is needed to clarify the role of fibrinogen in PD.

It is worth noting that the lower incidence of hypertension found in the PD group in the study may be related to the use of dopaminergic drugs. Dopaminergic medication, such as dopamine agonists, used to treat Parkinson’s disease can cause orthostatic hypotension (37). Besides affecting blood pressure, dopaminergic drugs may interact with antihypertensive medications (38). This study found higher alcohol consumption in the PD group, although existing research presents conflicting views on the impact of alcohol on PD. A prospective study from the United Kingdom reported that alcohol intake was associated with a higher risk of PD (39). Similarly, a large cohort study from Sweden suggested that heavy alcohol consumption increases the risk of PD in both women and men (40). In contrast, a Mendelian study found a positive correlation between genetic susceptibility to PD and alcohol consumption. Conclusions on the association between alcohol, coffee consumption, and PD are limited by insufficient statistical power (41).

In the subgroup analysis of this study, differences in NLR, LMR, and AFR were not significant when comparing groups based on disease duration and Hoehn–Yahr staging. We attribute this finding to the nature of PD as a chronic degenerative neurological condition, which induces long-term changes in the body’s immune response. Chronic inflammation due to long-term exposure leads to elevated levels of peripheral blood inflammation markers. This results in negligible differences in inflammation indicators across varying disease severities. Additionally, this study highlights differences in lipid levels between PD patients and healthy controls. Cholesterol’s effect on PD can be bidirectional, potentially protective or harmful. High cholesterol levels may trigger the accumulation of cholesterol in immune cells, leading to the release of pro-inflammatory cytokines, which can result in neuroinflammation (42). In a prospective cohort study, the association of lipids and apolipoproteins with future PD risk was investigated. Mendelian randomisation analyses confirmed that TC, LDL-C and triglycerides were causally and negatively associated with PD risk (43). Various clinical studies suggest that HDL and cholesterol may have a protective effect against the development of PD (44–46). These epidemiological findings support the conclusions of our study. Furthermore, research by Li and Liu indicated that high-density lipoprotein ratio (NHR) levels were significantly higher in PD patients than in healthy controls, and these levels negatively correlated with disease duration. Compared to the monocyte-to-high-density lipoprotein ratio (MHR) and NLR, NHR might be a more reliable predictor of long-term clinical outcomes in PD patients (47, 48). In conclusion, NLR, LMR, and AFR are associated with the severity of PD.

This study has several limitations. On the one hand, the participants’ data were extracted from a single medical center, and the sample size needs to be further expanded. On the other hand, NLR, MLR, and AFR, which are relevant indicators of systemic immune inflammation, could be affected by other factors. We cannot accurately draw conclusions about their specific effects on certain neuroinflammatory pathways. Therefore, the association between these inflammatory parameters and Parkinson’s disease risk needs further validation through multicenter studies with larger sample sizes.

In summary, chronic neuroinflammation is a well-established factor that negatively impacts the central nervous system (6). More specific biomarkers are required for clinical management, including evaluation and prediction. Identifying risk factors and biomarkers for Parkinson’s disease can elucidate the disease mechanism and improve prevention, treatment, and post-treatment management.

## Data Availability

The raw data supporting the conclusions of this article will be made available by the authors, without undue reservation.
